# Treasure of the Past: III: Gravitational Anisotropy in Crystals

**DOI:** 10.6028/jres.105.051

**Published:** 2000-08-01

**Authors:** Paul R. Heyl

## Abstract

Einstein’s theory of gravitation is based upon a fundamental postulate which asserts that gravitation and inertia are identical in nature and hence indistinguishable. This if true, is of the greatest theoretical importance, for gravitation has heretofore refused to show any relationship to other physical phenomena.

A most delicate test of this postulate is possible in a crystal of one of the nonisometric systems; for in such a crystal every known physical property (except inertia and, possibly, weight) varies with the axial direction in the crystal; and it is an interesting question whether, in such a crystal, gravitation will be found to align itself with inertia or will show some variability which will classify it with the great majority of physical phenomena.

To test this point, large crystals were weighed in different axial positions with respect to the earth. The specimens examined represented all five nonisometric systems, and were weighed to a precision, in most cases, of 1 part in 10^9^. The results were uniformly negative, and to this degree of precision are in Einstein’s favor.

Incidentally, this work has shown the practical possibility of using the gravity balance to a precision of I part in 10^9^, even when the swing of the beam must.be stopped and the object turned through a considerable angle. A precision of about the same order was attained by Majorana in 1920. In this work it was not necessary to arrest the beam or to touch the load. The next best record (in work of a somewhat different kind) at the International Bureau of Weights and Measures is 7 parts in 10^9^.

## I. STATEMENT OF THE PROBLEM

Einstein’s principle of equivalence, the fundamental postulate upon which is based his theory of gravitation, asserts in effect that gravitation and inertia are identical in their nature, and hence indistinguishable. The importance recently assumed by the Einstein theory of gravitation requires a careful examination of this underlying postulate.

This postulate, of course, was suggested by certain experimental results. It has long been known that the attraction of gravitation is independent of the nature of the substance, or, in other words, the inert mass of a body is proportional to its gravitative mass. Newton’s pendulum work^1^ established this roughly; Bessel,^2^ by similar but more refined experiments, reached a precision of 1 part in 60,000; and Eötvös,^3^ with his torsion balance, reached an accuracy of I part in 20,000,000. The latest work on this question, by Eötvös, Pekar, and Pekete,^4^ establishes this proportionality to 6 parts in 10^9^, nearly I0 times the precision of the earlier work of Eötvös. The substances examined were water, copper, platinum, magnalium, copper sulphate (solid and solution), asbestos, and talc.

The most promising place to look for a difference between gravitation and inertia would seem to be in a crystal of one of the nonisotropic systems. It is generally assumed (with, it must be admitted, no very precise experimental basis) that the inertia of such a crystal is independent of the axial direction; and in this respect inertia is unique, for every other investigated property of such a crystal (except, perhaps, weight) varies with the axial direction. Refractive index, polarization, selective absorption, thermal conductivity, pyro-electric and piezo-electric phenomena, electrical conductivity, elastic constants, and even the hardness of the different faces all show this axial difference; and it is a f air question whether a crystal, otherwise anisotropic, is gravitationally so; that is, whether gravitation will behave as inertia is supposed to behave or show some variability, as does every other physical property of crystals.

## II. PREVIOUS WORK

The possibility that crystals may exhibit a gravitational anisotropy has not gone unrecognized, though but little work has been done on the subject, and all of it antedates Einstein. Mackenzie^5^ compared the attraction of calcite crystals for similar crystals and for noncrystalline bodies at small distances by means of a torsion balance, and obtained negative results of a precision, in the highest instance, of 1 part in 900. Poynting and Gray^6^ repeated this work with quartz, using a method depending upon torsional vibration, also obtaining negative results, the precision reaching I part in 16,000. To these references may be added some unpublished work on quartz, by direct weighing, by Dr. S. W. Stratton, formerly director of the Bureau of Standards, also with negative results. It may be noted that all this work has been confined to crystals of the hexagonal system.

## III. GENERAL PLAN OF THE EXPERIMENTS

The experiments about to be described were planned to include specimens of all the nonisotropic systems of crystals. The method was that of direct comparison of weights in different axial positions. The crystal was mounted on the balance pan with, say, its *a* axis vertical, and counterpoised. The resting point of the balance was then determined. The crystal was then turned to bring, say, its *b* axis vertical, and the resting point redetermined. The crystal was turned again, in the same direction, until the *a* axis was again vertical, but, of course, in the reverse of its former position, and the resting point again noted. These operations were repeated, always turning the crystal in the same direction. Any three consecutive determinations of the resting point constitute what we shall call a weighing, the second value being compared with the arithmetical mean of the first and third. This, in outline, constitutes the method of procedure. Various precautions must, of course, be taken to insure results of precision.

## IV. SOURCES OF ERROR

### 1. ELONGATED FORM

In the case of a much elongated body, whether crystal or otherwise, a small differential effect, arising from purely geometrical considerations, may be expected as between the vertical and horizontal positions. A simple calculation shows that in the extreme case of a rod 20 cm long, with its mass concentrated in equal portions at its ends, the difference in weight is of the order of 10^−15^.

A paper by Mugge^7^ discusses similarly the case of a body made up of elongated particles similarly oriented, and finds the difference in weight to be not greater than I part in 10^−15^. A difference of this order is far beyond experimental reach, and is not of the nature of what is here meant by gravitational anisotropy.

### 2. HEIGHT OF CENTER OF GRAVITY

Much more serious differences in weight may arise from variations in the height of the center of gravity of the crystal when turned into its different positions. An error of 1 part in 10^9^ of the weight may result from a difference in height of the center of gravity as small as 3 mm. Such a difference in weight is readily distinguishable from an effect resulting from gravitational anisotropy; for, in the former case, as the crystal is turned continually in the same direction, the variation in weight will pass through a complete cycle in one revolution of the crystal; while in the case of gravitational anisotropy the cycle should be complete in half a revolution.

### 3. BUOYANCY

Apparent differences in weight are also to be looked for if there is, during any weighing, a variation in the density of the air, either from change of temperature or change of pressure. As most of the crystal specimens used were too bulky to be accommodated in the bureau’s vacuum balance, an alternative plan was adopted, that of counterpoising the crystal and its supporting frame by material of equal weight and volume. The precision with which this can be accomplished is, as we shall later see, ample.

### 4. ELECTRIFICATION

The question of possible electrification of the crystal is also to be considered. Such an occurrence might cause, in the different positions of the crystal, a differential attraction for the floor of the balance case which would simulate the effect of a gravitational variation. An observed positive result would, therefore, require checking in the presence of radium in the balance case. The uniformly negative results rendered this unnecessary.

### 5. CRYSTALLINE INERTIA

There is also to be considered the fact, earlier mentioned, that the assumption of uniform inertia in the direction of the different crystalline axes rests upon no precise basis of experiment. It is conceivable that a positive gravitational difference might be found without invalidating the principle of equivalence; for the inertia might differ slightly in the same proportion. This point would then require further investigation by means of the torsion balance, after the method of Eötvös. Here, again, the uniformly negative results make such a variation in inertia highly improbable.

### 6. TEMPERATURE GRADIENT

Passing from these relatively harmless sources of error, we find two factors which may seriously interfere with precision weighing. The first of these is the presence of small temperature differences within the balance case. The balance beam has a coefficient of expansion of the order 10^−5^, and if the two halves of the beam differ in average temperature by one ten-thousandth of a degree an error of 1 part in 10^9^ is introduced into the weighing. Of course, if this temperature gradient would remain constant the error would vanish; but this can not be depended upon. Should there arise for any reason a temperature difference of 0.1° between opposite walls of the weighing room (3 m apart) in which the balance case is set up, it is reasonable to expect the corresponding sides of the balance case to respond at least to 0.001 °, producing an error of 1 part in 100,000,000. Such small temperature changes are practically beyond control. The best practical solution is to surround the balance case with a heat-insulating cover, and then to watch and wait for the most favorable times for observation.

### 7. BEAM ARREST

**Fig. 1:**
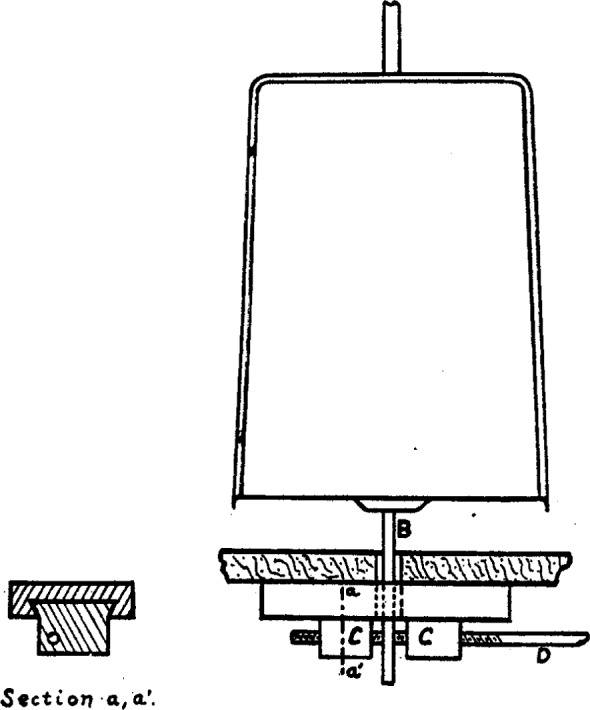
Poynting clamp.

A second serious source of error is introduced by the ordinary method of arrest and release of the beam, namely, by raising and lowering the central knife-edge bearing. The beam can not be depended upon to assume exactly the same state of flexure twice in succession; neither can we be sure that the knife-edge will always come down in exactly the same place. Poynting^8^ 45 years ago invented a device intended to meet this difficulty, which seems to have been very little used since. (Fig. 1.)

Attached rigidly to the bottom of the scale pan carrying the crystal and running downward through a hole in the floor of the balance case is a polished steel rod *B*, 3 mm in diameter. Fastened to the under side of the floor is an arrangement consisting of two sliding jaws of brass *C, C*, actuated by a steel rod *D*, carrying a right and a left hand thread. Between these jaws the vertical rod from the pan hangs freely. Its central adjustment, which is important, is made possible by sliding the threaded rod *D*, and with it the brass jaws *C, C*, slightly to the right or left and fixing it in position by lock nuts where the rod passes out of the balance case. The rod passes out through a piece of brass tubing inserted in the wall of the balance case, and against the ends of this tubing the lock nuts are adjusted. The lock nuts are set so as to prevent lateral motion, but not so tightly as to hinder the rod from turning. When in adjustment, the jaws clamp the vertical rod without tilting it and hold the scale pan motionless. Any weight on this pan may now be changed, or the crystal may be turned without altering the state of flexure of the beam, which is maintained constant by the counterweight in the other pan. Moreover, no change takes place in the position of the knife-edge.

This device was used by Poynting for the comparison of weights by the method of substitution and deserves a more extended recognition than it has obtained. In the present series of experiments it has proved itself an indispensable aid in reaching results of the highest precision. Freed from the limitation set upon its precision by the usual method of arrest and release, the workable sensitiveness of the instrument is capable of an increase of between 10 and 20 times that at which it is usually rated, and is limited, apparently, only by the presence of small and variable temperature differences within the balance case.

It will be of interest at this point to give figures showing the comparative behavior of the balance, using the ordinary beam arrest, and with the Poynting clamp. The sensitivity on this occasion was such that 0. 1 mm on the scale was equivalent to a change in weight of 1 part in 10^8^. Higher sensitivities were used in all the actual crystal weighings.

It will be seen that the individual readings with the Poynting clamp agree to 0.1 mm, the limit of reading, and that the average departure from the mean is about one-seventeenth that of the other series.

The conditions specifying the sensitivity usually employed were as follows:

Distance mirror to telescope...................cm.. 200
Distance, scale to mirror......................cm.. 200
Scale of millimeters...................photographed natural size
Magnifying power of telescope...........diameters.... 8

To reach a sensitivity of 1 part in 10^9^ the balance was adjusted so that a change in weight of 1 mg in a load of about 1,300 g produced a shift of about 8 cm in the field of view of the telescope, or an angular motion of the reflected ray of about 0.04 radian. The angular motion of the balance beam itself was, of course, 0.02 radian, and its time of half swing about 90 seconds.

The possibility exists of reaching a still higher precision without lengthening the time of swing by increasing the distance from mirror to scale, with, of course, a corresponding increase in magnifying power of the telescope; or by the use of a bifilarly supported mirror, such as used by Poynting; or by the use of certain recently developed optical lever systems, such as described by Tuckerman in Engineering, August 17, 1923, and by Gehlhoff in Zeitschrift für Technische Physik, No. 6, 1922. In this connection it may be recalled that Majorana (Phil. Mag., vol. 39, p. 488, 1920) reached a sensitivity of about the same order, but under conditions impossible to attain in work of the kind described in the present paper. In Majorana’s work it was not necessary to alter or even touch the weight or the balance, and the experiments were carried out in a vacuum. Moreover, the distance from telescope to scale was six times that used in the present investigation.

## V. DESCRIPTION OF APPARATUS

The balance used was by Rueprecht of Vienna, with a 30 cm beam, and a rated capacity of 2 kg in each pan. It was provided with attachments by means of which the fractional-gram weights could be handled without opening the case. The balance was set up in a small weighing room located in the basement of the electrical building of the bureau, and the various operations connected with arrest, turning the crystal, and re lease were conducted from outside the room by connecting rods.

**Fig. 2:**
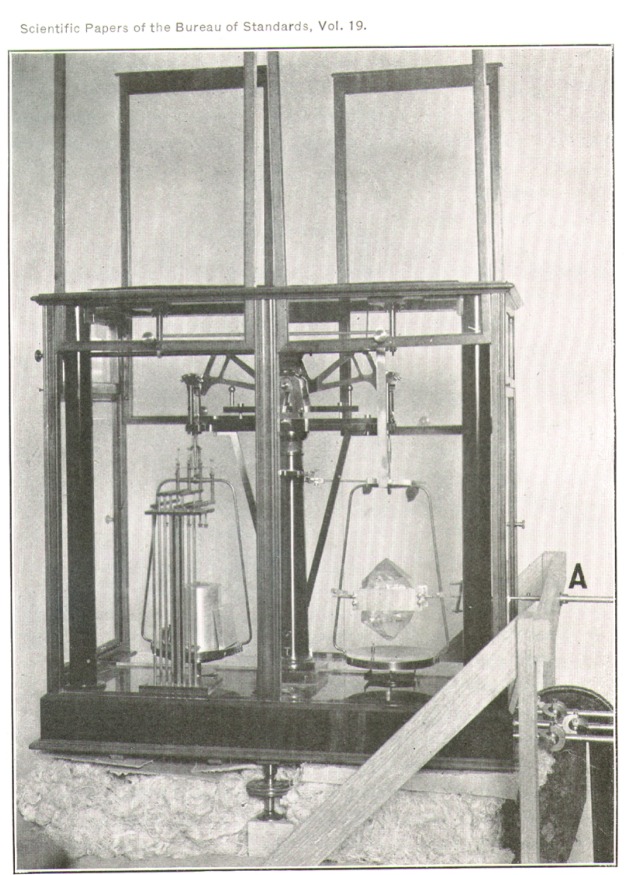
General view of apparatus.

Figure 2 shows a general view of the arrangement. The balance is clamped firmly to the top of the pier with small blocks under its leveling screws, so as to leave a space of about 10 cm between the bottom of the balance case and the top of the pier. This space is packed with cotton waste as a part of the heat insulation around the balance case.

**Fig. 3:**
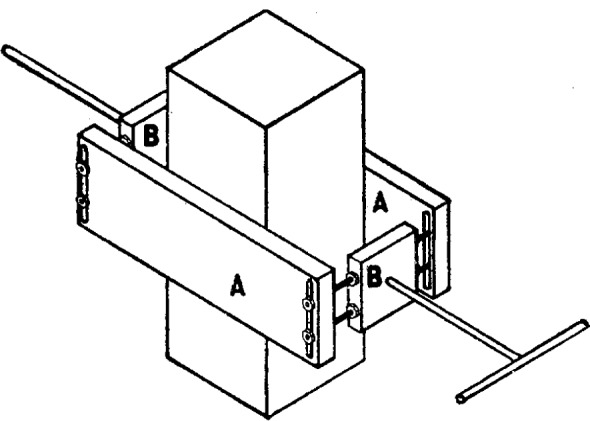
Frame for hilding crystal.

A large topaz crystal may be seen in position in the right-hand pan. The crystal is mounted in a clamping frame and can be turned about a horizontal axis by means of the uppermost of the four connecting rods seen in the figure. A more detailed view of this arrangement is shown in Figure 3.

**Fig. 4:**
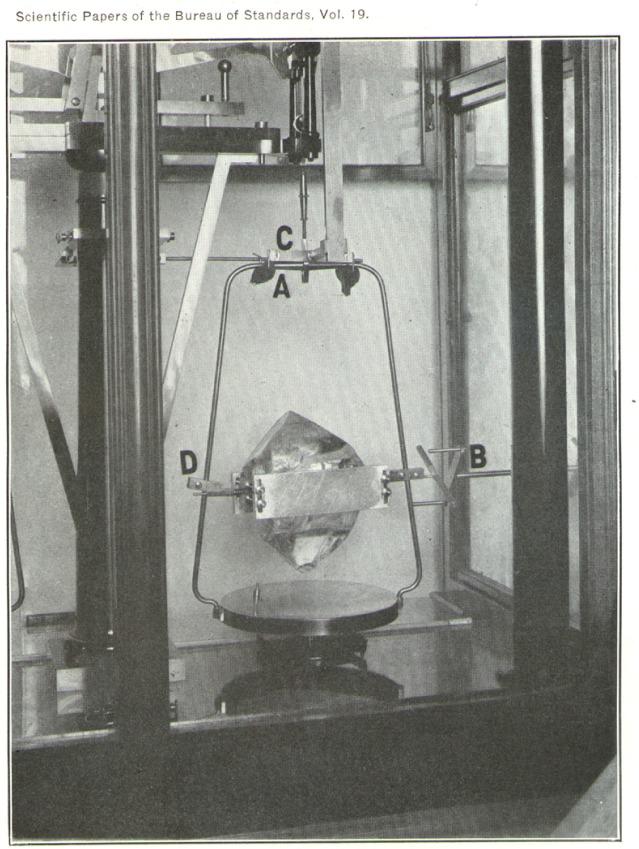
Detail view of crystal mounting and turning device.

The crystal is held between two flat pieces of aluminum (*A*, fig. 3) 10 cm long, 3 cm wide, and 0.8 cm thick. To accommodate natural irregularities of the crystal and to prevent injury to the specimen, pads of tin foil are placed between the crystal and the.clamps. The clamps are held together by two rods of brass at each end, passing through slots in the aluminum clamping pieces and provided with nuts by means of which the clamping action on the crystal is secured. These brass rods are threaded along their whole length, and upon each pair there slides freely a rectangular brass block (*B*, fig. 3), which may be fixed in any desired position along the rods by four nuts. Each brass block carries a projecting rod to serve as a support for the crystal, and the right-hand support is provided at its end with a crosspiece for turning the crystal. These supporting rods, when the crystal is in place, rest in brass gutters clamped to the side pieces of the frame of the scale pan (*D*, fig. 4). These gutters are placed on alternate sides (front and back) of the side pieces of the frame to permit the center of gravity of the crystal to be brought over the center of the pan. For this purpose, a few millimeters play, right and left, is permitted the supports in the gutters, and when this adjustment is obtained the free part of each rod between block and gutter is wound with a few turns of heavy copper wire as a washer to maintain this adjustment.

The end of the upper connecting rod is provided with a fork (*B*, fig. 4) which engages with the crosspiece on the crystal support when the crystal is turned. It is not always possible to have both arms of the fork engage with the crosspiece simultaneously, and in order to hold the scale pan against the twist that may result another clamping device is added. Near the top of the frame of the scale pan is mounted a fork (*C*, fig. 4), whose arms are provided with felt pads. As controlled from outside by one of the connecting rods, these padded arms may be lowered or raised and caused to press upward against the upper part of the frame by spring pressure, assisting the Poynting clamp in holding the frame while the crystal is being turned.

**Fig. 5:**
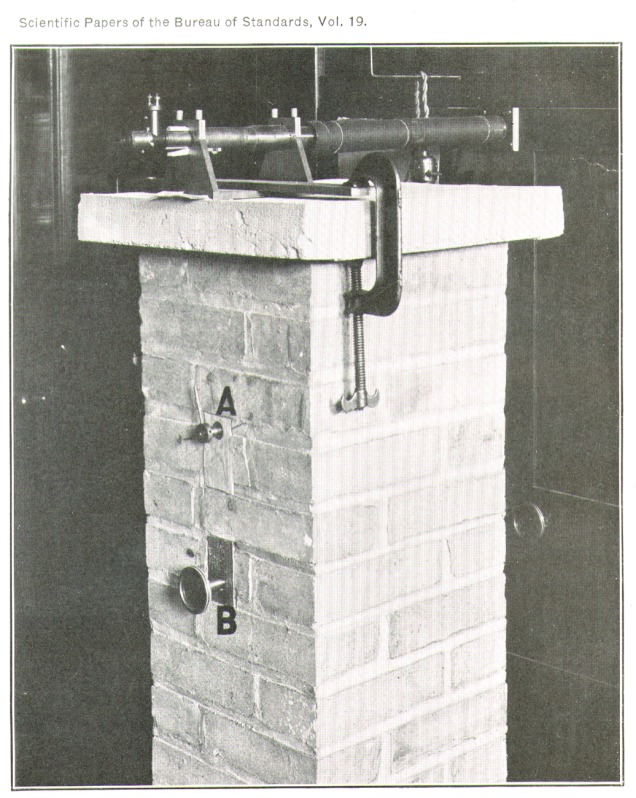
Outside control and observing apparatus.

To insure a smooth and regular motion in turning the crystal, the turning rod runs in ball bearings, one of which is located at *A* in the crosspiece of the wooden frame seen in Figure 2 and the other at the operating end of the rod, outside the weighing room. (A, fig. 5)

The various operations connected with turning the crystal are performed in the following order:

Set Poynting clamp as near the middle of the swing as possible.
Set upper holder (fork with padded arms).
Turn crystal.
Release upper holder.
Release Poynting clamp, usually slowly, but if additional motion is desired more rapidly.

To reduce as much as possible the entrance of air currents by the hole through which the steel rod passes through the floor of the case, two pieces of stiff paper, notched on their edges, are placed closely around the rod, covering the hole almost completely. By swinging the beam through a large angle these pieces of paper will be pushed away from the rod sufficiently to give ample clearance for the small swings used in weighing.

By means of the procedure just described a crystal may be weighed in any two axial positions. If it is desired to include a third axis in the comparison, it is necessary to remove the crystal from its clamp and alter its position so that a comparison may be made between the third axis and one of the first two.

The swings of the balance are noted in the usual way. A small mirror is affixed to the beam, and in the wall of the weighing room there is a transparent scale graduated in millimeters, illuminated by a lamp on the outside. The reflection of the scale is observed by a telescope mounted outside the weighing room on a brick pier. (Fig. 5.) On the front of this pier can be seen the knob, *B*, actuating the Poynting clamp, and above it the end of the crystal turning rod, *A.* The ends of the rods actuating the upper clamp and the usual beam arrest are on the wall of the weighing room behind the pier.

**Fig. 6:**
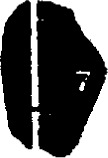
Appcarance of scale and cross uire.

The scale used was a photographic negative of a scale of millimeters. Its appearance is shown in Figure 6. Across the broad white vertical band can be seen a portion of the cross wire of the telescope. A negative rather than a positive was used to reduce to a minimum the amount of light entering the balance case. Except for this, the weighing room was completely dark. This precaution was found necessary by experience. With a scale with a bright background and the balance set for a sensitivity of 1 part in 10^9^, it was found that the resting point would drift rapidly and irregularly for over an hour after the light was turned on. The substitution of a negative scale greatly reduced this trouble.

The scale was calibrated by a micrometer microscope. The greatest variation found in any single millimeter was 0.02, and as the readings were taken only to 0 1 this was entirely satisfactory.

The heat insulation of the balance was completed by surrounding the case on all sides and the top by slabs of cork 7 cm thick, leaving space only for the operating rods and for viewing the mirror. Over the whole was laid a blanket.

## VI. METHOD OF PROCEDURE,

In beginning work with a crystal specimen the first thing was to determine its mass and volume. These determinations were made by Mr. Peffer of the bureau’s volumetric section. The different specimens ranged in weight from 375 to over 1,300 g and in density from 4.560 (zircon) to 2.308 (gypsum). For each crystal a counterweight was constructed in the form of a cylinder equal in weight and in volume to the crystal. For the denser crystals the counterweight was made of aluminum with a brass plug; for the lighter crystals the aluminum cylinder was constructed with a hole closed at the top by a plug of aluminum fitting so tightly that it was necessary to use a large lever press to insert it. The counterweights were washed with gasoline and baked at 120° for 24 hours to remove the kerosene used in machining the aluminum. It was found possible to construct a counterweight in this way correct in volume to 0.2 cm^3^ or better, simultaneously with an error in weight of less than 0.05 g.

The crystal was then placed in its clamp support, record being made of the separate weights of aluminum, tin foil, and brass constituting the support. Equal weights of aluminum and tin foil were provided for counterpoising, the brass parts being counterpoised in the final adjustment by brass weights, fractional gram weights, and the rider.

The error, or differential buoyancy, resulting from this procedure is negligibly small. Such an error depends upon the changes in temperature and pressure to be expected during the 45 minutes required for a weighing. The greatest temperature change observed in this period was 0.1°. Such observations could not be made during a regular series of weighings, as the room had to be kept dark; but special observations for temperature were taken on other occasions when weighings were not in progress, with the above result. The greatest change in pressure was about 0.3 mm, as calculated from the daily record of the barometer on the bureau grounds. Acting together, changes of this magnitude would alter the density of a given mass of air by about 7 parts in 10,000.

The greatest differential volume in the case is that of the crystal and its counterweight, which as a maximum may reach 0. 2 cm^3^. The next largest is that arising from the steel rod below the pan, balanced by brass weights. This amounts to about 0.08 cm^3^. The last item arises from fractional gram weights of platinum and aluminum against brass (principally). In the extreme case of fractional weights aggregating 1 g, all of platinum, against brass the differential volume is about 0.07 cm^3^. A total possible, but improbable, volume results of 0.35 cm^3^. This volume of air would weigh about 0.4 mg. The increase in buoyancy to be expected for a change in density of 7 parts in 10,000 would be about 0.0003 mg or about 1 part in 3 × 10^9^ of a kilogram crystal, about one-third the smallest quantity weighable in the experiments.

The next adjustment was to bring the center of gravity of the crystal sufficiently near the axis about which it was to be rotated. For this purpose the crystal in its support was placed on knife-edges. A rough adjustment was made by shifting the crystal in its clamp, and the final adjustment by moving the axles. It will be recalled that two degrees of freedom were provided for these axles—one by sliding the blocks along the threaded side rods, another by moving the rods themselves in the slots in the aluminum clamping pieces. With crystals of the minimum weight employed (375 g) it is easy to effect an adjustment to a fraction of a millimeter, for with a mass of this magnitude an error of a single millimeter in balancing will produce a torque of about 37 g-cm; and, as before stated, the accuracy of adjustment required for a precision of 1 part in 10^9^ is about 3 mm.

The crystal was now placed in position on the scale pan and counterpoised. The Poynting clamp was then adjusted so that the rod from the scale pan should hang vertically and centrally between the two brass jaws. This adjustment is made by a somewhat tedious matter of trial, and is greatly facilitated by having a weight of about 20 g in the form of a thin pad of tin foil on the scale pan carrying the crystal, with, of course, a counterpoising pad on the other pan. By moving this weight to right or left the verticality of the rod may be exactly adjusted. The central position between the jaws must be attained by shifting the threaded rod which actuates the jaws by means of the lock nuts where the rod passes through the balance case. On setting the clamp while looking through the telescope the jolt or shift of the scale should be a minimum. With the mirror at a distance of about 200 cm from the scale and telescope it was found possible to reduce this shift to about 1 mm.

The sensitivity was next determined in the usual manner. No attempt was made to read the scale more closely than the nearest millimeter. Closer readings would have been worthless, as it was necessary to turn on the light and enter the weighing room in order to shift the rider, and the cork housing around the balance case had to be left partly open to effect this. An approximate value of the sensitivity is all that is required.

To reach sensitivities of over a hundred million it was necessary to load the balance beam above the point of support, as there was not sufficient travel of the bob on the pointer. This was accomplished by means of several riders of copper wire. The final adjustment was made by the bob on the pointer. With a load of 1,600 g it was found possible to reach a sensitivity such that 0.1 mm on the scale was equivalent to about 1 part in 2½ billion (2.5 ×10^9^) of the load before the beam became unstable. The time of half swing at this sensitivity was about two minutes. At this sensitivity the wandering of the zero was too great to permit of observations of any reasonable concordance. For most specimens it was found possible to work at a sensitivity of 1 part in 10^9^ of the weight of the crystal, but in the case of the monoclinic specimen (gypsum), which weighed only 375 g, it was necessary to work at half this sensitivity.

In working at the highest sensitivities it was noticed that the balance might be stable near the middle of its swing, but that if the swing was extended too far in either direction the beam became unstable. The explanation is perhaps to be found in (1) the reduced flexure of the beam when tilted, due to the fact that the bending forces are no longer perpendicular to the line joining the terminal knife-edges, and (2) in the very small distance originally between the center of gravity and the center of support, so small that it might be wiped out entirely by the reduction in flexure due to tilt. The same result might also be caused by a slight flattening of the central knife-edge.

At a sensitivity of 10^9^, with a load of 1,600 g in each pan, the time of half swing was about 100 seconds.

The desired sensitivity having been reached, the cork housing was replaced in position and the whole covered by a blanket. The weighing room was then darkened and left for several hours before an attempt was made to take readings.

A watch was kept from time to time on the resting point of the balance, which was left unclamped and free to swing, and when the drift seemed promisingly slow a weighing was attempted. Night readings showed, on the whole, no better results than those obtained by day, provided that the best part of the day was used. This proved to be from 1 to 3 p. m. Certain days could not be used at all; and, again, a rainy day following a rainy night could be used through all its hours.

## VII. RESULTS

The system followed in taking readings will be best explained by giving a complete numerical example, after which the results for the various specimens will be more compactly summarized.

That is to say, 0 1mm on the scale is equivalent to a change in weight of 1 part in 1.14 billion of the weight of the crystal, 1,300 g. As this value is only approximate, we may regard it as 1 part in 1 billion.

The following figures give the record for a complete weighing of the same crystal. The letters a, *b* under “position of crystal” indicate which crystalline axis was vertical.

A negative result with a precision of about 1 part in 10^8^.

**Fig. 7:**
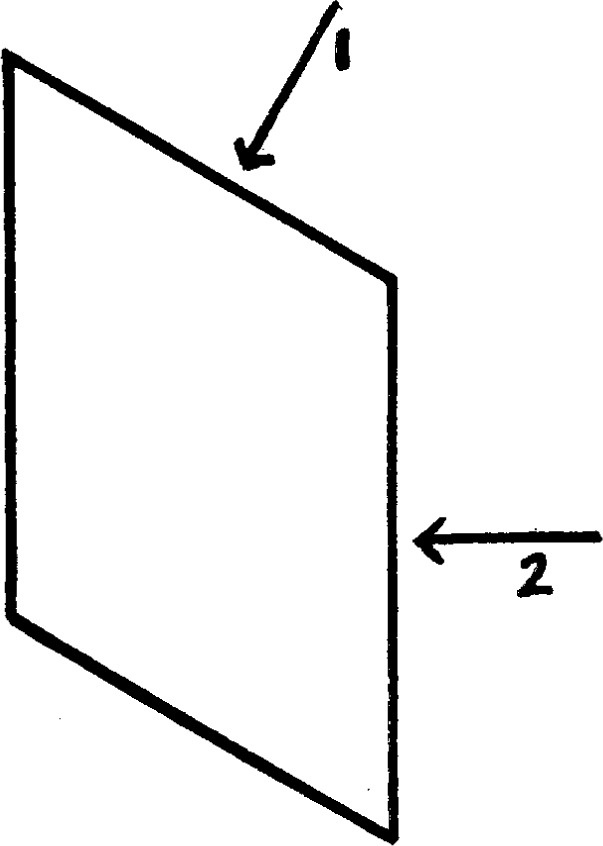
Axes of gypsum crystal.

**Fig. 8:**
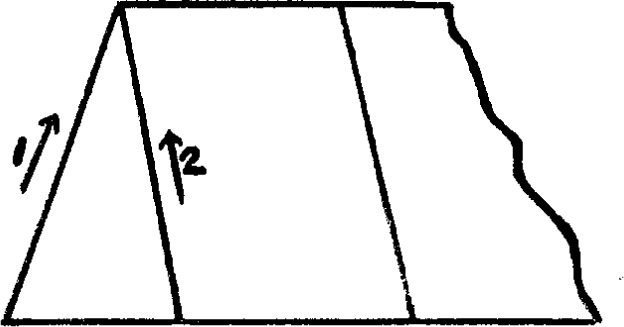
Axes of microcline crystal.

We may now summarize the results obtained with the various crystalline specimens. The accepted direction of the crystalline axes is rather conventional, especially in the monoclinic and triclinic systems, where the usual crystallographic axes do not coincide either with the optic axes or the natural axes of elasticity. There is no means of telling in which direction the gravitational axes would lie should such exist. The crystals were, therefore, oriented for weighing according to the usual crystalline axes. In the tetragonal and hexagonal systems but two positions were necessary, with the *c* axis vertical and horizontal, as the other axes were all equal and the crystal isotropic in their plane. In the orthorhombic system the three different axial positions were treated in pairs. In the monoclinic and triclinic systems the same was done. To avoid ambiguity in these latter cases the directions used are indicated in Figures 7 and 8. The third direction in each case is perpendicular to the paper.

Particular care was taken to select crystal specimens that were not twinned and were as fine individual specimens as possible. In this connection mention must be made of the courtesy of the Smithsonian Institution, which, through Dr. G. P. Merrill, placed at the disposal of the Bureau of Standards the large collection of minerals in the National Museum.

All results are negative to the degree of precision indicated. Each series of weighings mentioned, 25 or 30 in number, is complete and continuous; that is, no intermediate weighings were discarded for any reason, low precision or otherwise. Weighings were made over a period of several weeks, sometimes one a day, sometimes more, as conditions permitted. Before attempting a weighing the drift of the resting point was examined, and if this appeared unpropitious no weighing was attempted. If conditions appeared favorable, a weighing was begun and the result recorded, even though not always up to expectations. The only exception to this was in the case of the monoclinic system (gypsum), which presented unusual difficulty from the light weight of the specimen and a slight hygroscopic tendency. Here the series is indeed continuous, but after its termination as here recorded a number of weighings were obtained which were discarded, as their precision showed an increasing tendency for the worse.

### 1. TETRAGONAL SYSTEM

#### Zircon

Weight, 1,337 g Weighed in two positions, with *c* axis vertical and horizontal. Weighings obtained, 30, of which 14 reached a precision of 1 part in 10^9^. Average precision of all the weighings, 3 parts in 10^9^.

### 2. HEXAGONAL SYSTEM

Two specimens from this system were examined: Quartz and tourmaline, the latter chosen on account of its unusual anisotropic character. Tourmaline is dichroic, polarizes by absorption, and is both pyro-electric and piezo-electric. Both specimens were weighed, as in the tetragonal system, in two positions, with the *c* axis vertical and horizontal.

#### Quartz

Weight, 648 g. Weighings obtained, 31, of which 9 reached a precision of 1 part in 10^9^. Average precision, 4 parts in 10^9^.

#### Tourmaline

Weight, 1,207 g Weighings obtained, 30, of which 11 reached a precision of 1 part in 10^9^. Average precision, 2.5 parts in 10^9^.

### 3. ORTHORHOMBIC SYSTEM

#### Topaz

Weight, 1,295 g Weighed in three positions.

Axes *a* and *b.* Weighings obtained, 31, of which 8 reached a precision of 1 part in 10^9^. Average precision, 4 parts in 10^9^.

Axes *b* and *c.* Weighings obtained, 36, of which 11 reached a precision of 1 part in 10^9^. Average precision, 3 parts in 10^9^.

### 4. MONOCLINIC SYSTEM

#### Gypsum

Two crystals were weighed together, total weight, 375 g Because of this small weight the balance was unusually sensitive to minute air currents. The crystals also seemed slightly hygroscopic. A slow but continual increase in weight was noted, lasting for four weeks (the whole period of the weighing) and varying somewhat in rate. The average increase was about 0.005 g Per week. Because of these two difficulties it was necessary to reduce the working sensitivity of the balance to 2 parts in 10^9^; and even at this reduced figure it was found impossible to obtain as high a percentage of weighings of the maximum precision as with the other crystals.

The specimens were weighed in three positions, indicated in Figure 7, the third position being perpendicular to the paper.

##### Positions 1, 2

Weighings obtained, 24, of which 3 reached a precision of 2 parts in 10^9^. Average precision, 12 parts in 10^9^.

##### Positions 1, 3

Weighings obtained, 30, of which 5 reached a precision of 2 parts in 10^9^. Average precision, 12 parts in 10^9^.

### 5. TRICLINIC SYSTEM

#### Microcline

Weight of crystal, 837 g. Axial positions as indicated in Figure 8.

##### Positions 1, 3

Weighings obtained, 25, of which 7 reached a precision of 1 part in 10^9^. Average precision, 4 parts in 10^9^.

##### Positions 2, 3

Weighings obtained, 28, of which 4 reached a precision of 1 part in 10^9^. Average precision, 6 parts in 10^9^.

With this crystal much trouble was experienced from an apparent loss of weight on the part of the crystal, which began to manifest itself toward the end of the first set of weighings and continued throughout nearly all of the second set. For this reason the percentage of first-class weighings with this crystal is rather low.

## VIII. SUMMARY AND CONCLUSION

Six crystal specimens, representing the five nonisotropic crystalline systems, were weighed in different orientations in the earth’s gravitational field.

In all cases except one (gypsum) a precision of 1 part in 10^9^ was attained. In the case of gypsum the precision reached was 2 parts in 10^9^. To this degree of precision no difference in weight was observed. To the degree of precision of these results, gravitation and (assumedly) inertia must be placed in one class, and all other known crystalline properties in another.

This result is significant in view of Einstein’s contention that gravitation and inertia are of the same nature.

It has been shown that the gravity balance possesses possibilities of greatly increased precision when relieved from the limitation set by the usual method of arrest by raising the beam.

## Figures and Tables

**TABLE 1:** Resting Point of Centimeters. [Jan 22, 1923. Load, 1,600 g.]

With beam arrest.	With Poynting clamp.
	
4.31	4.23
4.18	4.22
4.31	4.23
4.17	4.24
4.32	4.23
Mean………………………… 4.26	4.23
Average departure from mean…. 0.07	0.004

**TABLE 2:** Sensitivity, March 12, 1923. [Topaz crystal (orthorhembic); weight 1,295.6 g; total load, about 1,600 g.]

Rider.	Scale reading in centimeters		Resting point.
		
	Up.	Down.	
1.0………………………………………………………………………	13.1		
		4.1	8.0
	10.5		7.7
		5.4	7.7
	9.2		7.6
		6.3	7.7
	8.7		7.7
		6.8	
			7.7
0.5……………………………………………………………………….		2.2	
	3.6		3.0
		2.4	3.0
	3.4		3.0
		2.6	3.0
	3.2		2.9
		2.6	2.9
	3.1		
			3.0
	7.4		
1.0……………………………………………………..		6.4	6.9
	7.3		6.9
		6.6	7.0
	7.3		7.0
		6.8	7.1
	7.3		7.1
		6.9	
			7.9
0.5…………………………………………………………………………	4.1		
		0.6	2.2
	3.4		2.2
		1.1	2.2
	2.9		2.1
		1.4	2.1
	2.7		2.1
		1.6	
			2.2
1.0………………………………………………………………………….	6.7		
		5.3	6.0
	6.6		6.1
		5.7	6.2
	6.7		6.3
		5.9	6.3
	6.5		6.3
		6.1	
			6.2

**TABLE 3:** 

Resting point.		Difference (0.5 mg).	Sensitivity.
		
Up.	Down.		
7.7			
	3.0	4.4	1.14×10^9^
7.0		4.4	1.14×10^9^
	2.2	4.4	1.14×10^9^
6.2			1.14×10^9^

**TABLE 4:** March 14, 1923. Same crystal and total load.

Time.	Readings in Centimeters.		Resting point.	Position of crystal.
			
1.30 p.m………………………………………………………………………	Up.	Down.		
	4.68			
		5.58	5.16	*a*
	4.80		5.17	
		5.49	5.17	
	4.88		5.17	
		5.40	5.16	
	4.94			
			5.17	
1.42………………………………………………………………………	5.44			
		4.95	5.19	*b*
	5.40		5.19	
		5.00	5.20	
	5.38		5.20	
		5.03	5.20	
	5.33			
			5.20	
1.52 p. m………………………………………………………………………	4.89			*a*
		5.52	5.23	
	4.97		5.24	
		5.48	5.24	
	5.02		5.24	
		5.44	5.25	
	5.09			
2.03 p.m………………………………………………………………………			5.24	

**Table:** 

		cm
Average resting point	*a.......*	5.21
	*b*.......	5.20
Difference..........		0.01

## Footnotes

1 Prindpia, Book III, Prop. 6, Theorem 6.

2 Pogg. Annalen, 25, p. 401; 1832.

3 Math, und Naturwiss. Berichte aus Ungarn, 8; I890; Beiblatter, 15. p. 688; 1891.

5 Physical Review, 2 P.32x; 1895.

6 Phil. Trans. Roy. Soc. Ann., 192, P. 245; 1899.

7 Centralblatt f űr Mineralogie pp. 33–39; 1913.

8 Proc. Roy. Soc., 28, pp. 2–35; 1878. 187

